# Epidemiology and Treatment of Acute Promyelocytic Leukemia in Latin America

**DOI:** 10.4084/MJHID.2011.049

**Published:** 2011-10-24

**Authors:** E.M. Rego, R.H. Jácomo

**Affiliations:** 1 National Institute of Science and Technology in Stem Cell and Cell Therapy, Division of Oncology/Hematology, Department of Internal Medicine, Medical School of Ribeirão Preto, University of São Paulo; 2 Department of Internal Medicine, Medical School, University of Brasília, Brazil

## Abstract

Distinct epidemiological characteristics have been described in Acute Promielocytic Leukemia (APL). Populations from Latin America have a higher incidence of APL and in some geographic areas a distinct distribution of the PML-RARA isoforms is present. Here, we review the main differences in APL epidemilogy in Latin America as well as treatment outcomes.

## Introduction

Geographical variations in frequency and clinical characteristics of hematological malignancies in patients from Europe/US and those from Latin America have been described in lymphoid malignancies,^1^–^4^ and to a lesser extend in myeloid neoplasms.^5^–^8^ Acute Promyelocytic Leukemia (APL) is a distinct type of myeloid leukemia characterized by its invariable association with chromosomal translocations involving the *Retinoid Acid Receptor α*(*RARA*) gene in chromosome 17. The breakpoints in *RARA* are clustered in relatively short span in intron 3. Likewise, the breakpoints in the *Promyelocytic Leukemia* (*PML*) gene, the main partner of *RARA* in APL-associated rearrangements, are clustered in three regions: one in exon 3 originating the short form type of *PML-RARA* fusion gene, or bcr3, and two in exon 6: the long form, or bcr1 and the variable form, or bcr2.^9^ Due to the fact that the *PML-RARA* is detected in more than 98% of patients with APL, the disease is a paradigm of acute myeloid leukemia (AML) associated with recurrent chromosomal translocations. In addition, APL is also distinct from other AML subtypes for its response to the all *trans* retinoic acid (ATRA), which induces terminal granulocytic differentiation of the blasts. Consequently, the combined use of ATRA and anthracyclines has become the mainstay of APL therapy and several trials reported cure rates above 80%.^10^

Despite the great improving in APL treatment, very few is known on the outcome of APL patients and their characteristics in developing countries, mostly due to absence of well structured networks that could permit sharing experiences and accrual of large number of patients. Only recently, the International Members Committee of the American Society of Hematology proposed the creation of a group that would create the necessary integration for collaboration in developing countries in an effort named International Consortium on Acute Promyelocytic Leukemia.

## APL Characteristics in Latin America

The data available on specific characteristics of APL patients on Latin America are from reports by single or few centers. Population based information is unavailable, due to inaccurate registries. Douer D et al^11^ were the first to report specific features of APL in patients with ‘latin’ and ‘non latin’ ancestry. Data from a single institution as well as a population-based study suggested that the Latino population had a higher proportion of APL among all AML diagnosis, which reached 37.5% against 6.4% in the non-Latino population. Nevertheless, Latino is not an ethnicity and is very difficult to define what is the characteristic of such population. The authors define as those originating in Latin America, but the genetic background of people from different areas of this vast region is very different. Spanish, Portuguese, Italian, African and others are present at different proportions with the native Americans which, by themselves vary as much as Mayan, Aztecs and the different Amerindians tribes from Brazil. Ruiz-Arguelles, GJ^12^ suggested that Latino should be considered as those speaking languages derived from Latin, but this also implies in very imprecise characterization, putting together populations so different as French, Italians, Romanians, Brazilians and Uruguayans among others.

No matter definitions, there is sufficient data on medical literature to support the fact that distinct populations have different incidences of APL. We have reported that APL represents 28.2% of all AML cases in centers in Brazil, a number that is very similar to the reported by Melo, R et al (28%).^13^ This is ratified by information from Mexico (20%), 12 Venezuela (27.8%)^14^ and Peru (22%).^15^ This is also true among children, despite the fact that APL is less common in this age group.^16^ The reason for this high incidence is to be explored. One may argue that in countries with difficult access to health support the young would have priority and, as APL has higher incidence in young adults, it would falsify the true incidence of other AML. However, a study conducted in Spain have also demonstrated a variable incidence of APL in different regions of the country.^17^ While the Northern region had relative incidence of 12.6%, in the Southern it was 21.6%. Another study from Spain has reported 23% of incidence of APL.^18^ Studies from Italy suggest an incidence of 11.5%, ranging from 27.7% in patients between 15 and 24 age years and 2.7% in older than 75 years.^19^ Since well controlled epidemiological studies carried out in UK^20^ and Scandinavian^21^ countries indicated that APL represented about 10% of all AML cases, one may argue that the incidence is lower in Northern Europe compared to Mediterranean countries, and in consequently in their former colonies in America.

Apart from the issue of incidence, some peculiarities regarding the clinical features have been described in patients from Latin America.^22^ In a survey evolving 12 Brazilian institutions that treat hematologic malignancies we observed that the mean age of diagnosis (36 years) and gender distribution (male 45.8% and female 54.8%) did not differ from the observed in literature.^6^ Furthermore, we noticed that there was a high incidence of the high relapse risk group as defined by the PETHEMA and GIMEMA groups.^23^ In our report, 36.9% of the patients had a white blood cell (WBC) counts above 10,000/μL contrasting with 22.6% of the combined Spanish and Italian experiences (p<0.001). This excess of high-risk patients may be secondary to difficult access to health assistance. It is worth noting that we did not detect differences in the distribution among PML breakpoint clustering regions (bcrs), with 54.3% of patients harboring the bcr1 isoform.^6^ This is relevant because bcr3 cases have been associated with higher WBC counts.^24^ Douer D et al reported a proportion of 75% of the bcr1 isoform of PML-RARA higher than the usual 50–55% reported in clinical trials.^23^,^25^ Further data is not homogeneous. A population of Mexican Mestizo APL patients showed 63% of bcr1 and in a population from the Northeast of Brazil there were 68.8% of bcr1/bcr2. This relative incidence is very similar to the reported in China (67%)^26^ and a combined Uruguayan and Argentinean report (62%).^27^

## APL Treatment in Latin America

The majority of centers in Latin America adopt protocols based on ATRA plus anthracyclines, similar to those reported by the Italian GIMEMA or the Spanish PETHEMA groups. However, a major concern is the cost of treatment. To supplant the high cost of idarubicin, many hospitals in Brazil adapt the protocol to another anthracycline such as daunorubicin^28^ or mitoxantrone.^29^ In our 12 center survey,^6^ we observed that three centers used distinct protocols not based on antracyclines and ATRA. Two used ATRA alone until remission and one of them used to consolidate with front line autologous bone marrow transplantation. A third one used a multiple drug protocol in induction.

Results from institutions that use AIDA similar regimens are not remarkable. In a single pediatric institution report, da Costa Moraes et al described that, among fourteen patients with APL diagnosis only five were discharged for follow-up.^28^ Pagnano et al reported similar results.^29^ In their report, in a seven-year period, out of 19 APL patients only eleven finished induction and eight finished the third consolidation cycle with a disease-free survival estimated of 82% at 120 months. This report raises an important issue in APL patients’ treatment. Death during chemotherapy was higher than in developed countries and the main causes of death were bleeding, infection and differentiation syndrome.

These results were corroborated by our larger survey analysing 134 patients treated with antracycline plus ATRA based therapy.^6^ Induction mortality was as high as 32.1% and bleeding was the cause of death in 60.5%. of the cases. Even during consolidation we observed a mortality rate of 10.5%, and mortality was mainly due to bleeding (21.4%), infection (28.6%) or their association (14.3%). The cumulative mortality was 44.7%.

The high incidence of high risk patients reported in the series from Brazil as well as the large mortality during treatment suggest that two main points should be addressed: expedite diagnosis and better support during treatment. This could be achieved with a high motivational effort and also with a network of specialists that could exchange experiences and create a well established protocol and unified support treatment guidelines. Previous efforts have shown that collaboration and educational programs can interfere with the outcome of cancer patients, specifically in children.^30^–^32^

APL is a very good candidate for collaboration. Treatment protocols are not complex and costs are not high. Furthermore, a high suspicious diagnosis is possible due to its characteristic morphology and bleeding tendency. The confirmatory diagnosis can be made in a few hours with an immunocytochemistry technique^33^ that requires only a fluorescence microscope and minimal training.^34^ Finally, the molecular characterization can be made in central laboratory with higher level of complexity.

Confronting the situation of APL in developing countries and the potential results that could be achieved the American Society of Hematology through its International Members Committee proposed a collaboration effort evolving developing countries named International Consortium on Acute Promyelocytic Leukemia (IC APL). Specialists in APL field from innumerous countries integrate the IC APL and nowadays the group runs a protocol in Brazil, Mexico, Uruguay and Chile. The protocol is similar to the PETHEMA 2005,^35^ but changing idarubicin to daunorubicin. We recently reported preliminary data from 97 patients included in the IC APL 2006 protocol.^36^ There was a remarkable improvement in survival that reached 75% in one year and the disease-free survival in the same period was 95%. Furthermore, there was an improvement in early mortality that we believe is mostly due to standardized prophylactic platelet an fresh frozen plasma transfusion.

The experience of IC-APL have shown that networking is an effective tool to improve medical assistance and infrastructure, as well as to perform clinical investigation in the setting of developing countries. Although preliminary, IC-APL data suggest that despite minor differences in the laboratorial differences exist, the clinical outcome of APL is similar once the early mortality is reduced by prompt diagnosis and effective supportive measures.

## References

[1] Pombo-de-Oliveira MS, Carvalho SM, Borducchi D, Dobbin J, Salvador J, Correa RB, Moellman A, Loureiro P, Chiattone C, Rios M (2001). Adult T-cell leukemia/lymphoma and cluster of HTLV-I associated diseases in Brazilian settings. Leuk Lymphoma.

[2] Pombo De, Oliveira MS, Loureiro P, Bittencourt A, Chiattone C, Borducchi D, De Carvalho SM, Barbosa HS, Rios M, Sill A, Cleghorn F, Blattner W (1999). Geographic diversity of adult t-cell leukemia/lymphoma in Brazil. The Brazilian ATLL Study Group. Int J Cancer.

[3] Gupta S, Bonilla M, Fuentes SL, Caniza M, Howard SC, Barr R, Greenberg ML, Ribeiro R, Sung L (2009). Incidence and predictors of treatment-related mortality in paediatric acute leukaemia in El Salvador. Br J Cancer.

[4] de Souza Reis R, de Camargo B, de Oliveira Santos M, de Oliveira JM, Azevedo Silva F, Pombo-de-Oliveira MS (2011). Childhood leukemia incidence in Brazil according to different geographical regions. Pediatr Blood Cancer.

[5] Ruiz-Arguelles GJ, Garces-Eisele J, Reyes-Nunez V, Gomez-Rangel JD, Ruiz-Delgado GJ (2004). More on geographic hematology: the breakpoint cluster regions of the PML/RARalpha fusion gene in Mexican Mestizo patients with promyelocytic leukemia are different from those in Caucasians. Leuk Lymphoma.

[6] Jácomo RH, Melo RA, Souto FR, de Mattos ER, de Oliveira CT, Fagundes EM, Bittencourt HN, Bittencourt RI, Bortolheiro TC, Paton EJ, Bendlin R, Ismael S, Chauffaille Mde L, Silva D, Pagnano KB, Ribeiro R, Rego EM (2007). Clinical features and outcomes of 134 Brazilians with acute promyelocytic leukemia who received ATRA and anthracyclines. Haematologica.

[7] Douer D (2003). The epidemiology of acute promyelocytic leukaemia. Best Pract Res Clin Haematol.

[8] Avvisati G, Lo Coco F, Diverio D, Falda M, Ferrara F, Lazzarino M, Russo D, Petti MC, Mandelli F (1996). AIDA (all-trans retinoic acid + idarubicin) in newly diagnosed acute promyelocytic leukemia: a Gruppo Italiano Malattie Ematologiche Maligne dell'Adulto (GIMEMA) pilot study. Blood.

[9] Pandolfi PP, Alcalay M, Fagioli M, Zangrilli D, Mencarelli A, Diverio D, Biondi A, Lo Coco F, Rambaldi A, Grignani F (1992). Genomic variability and alternative splicing generate multiple PML/RAR alpha transcripts that encode aberrant PML proteins and PML/RAR alpha isoforms in acute promyelocytic leukaemia. EMBO J.

[10] de la Serna J, Montesinos P, Vellenga E, Rayón C, Parody R, León A, Esteve J, Bergua JM, Milone G, Debén G, Rivas C, González M, Tormo M, Díaz-Mediavilla J, González JD, Negri S, Amutio E, Brunet S, Lowenberg B, Sanz MA (2008). Causes and prognostic factors of remission induction failure in patients with acute promyelocytic leukemia treated with all-trans retinoic acid and idarubicin. Blood.

[11] Douer D, Preston-Martin S, Chang E, Nichols PW, Watkins KJ, Levine AM (1996). High frequency of acute promyelocytic leukemia among Latinos with acute myeloid leukemia. Blood.

[12] Ruiz-Argüelles GJ (1997). Promyelocytic leukaemia in Mexican mestizos [letter]. Blood.

[13] Melo RA, de Vasconcellos JF, Melo FC, Machado CG, Lacerda TM, Souto FR (2006). PML-RARalpha fusion gene transcripts and biological features in acute promyelocytic leukemia patients. Clin Lab Haematol.

[14] De Salvo L, Weir Medina J, Gómez Sánchez O, de Baena ES, de Ramos BU, Guevara J, Luengo Vera J, de Vizcaíno MA, Sánchez H, de León E (1989). Acute promyelocytic leukemia in the west of Venezuela. Sangre (Barc).

[15] Otero JC, Santillana S, Fereyros G (1996). High frequency of acute promyelocytic leukemia among Latinos with acute myeloid leukemia. Blood.

[16] Mendes WL, Coser VM, Ramos G, Pereira W, Lopes LF, de Oliveira MS (2004). The apparent excess of acute promyelocytic leukemia in infant acute leukemias. Brazil Haematologica.

[17] Sierra M, Alonso A, Odero MD, Gonzalez MB, Lahortiga I, Perez JJ, Garcia JL, Gutierrez NC, Calasanz MJ, San Miguel JF, Hernandez JM (2006). Geographic differences in the incidence of cytogenetic abnormalities of acute myelogenous leukemia (AML) in Spain. Leuk Res.

[18] Tomas JF, Fernandez-Ranada JM (1996). About the increased frequency of acute promyelocytic leukemia among Latinos: the experience from a center in Spain. Blood.

[19] Pulsoni A, Stazi A, Cotichini R, Allione B, Cerri R, Di Bona E, Nosari AM, Pagano L, Recchia A, Ribersani M, Rocchi L, Veneri D, Visani G, Mandelli F, Mele A (1998). Acute promyelocytic leukaemia: epidemiology and risk factors. A report of the GIMEMA Italian archive of adult acute leukaemia. GIMEMA Cooperative Group. Eur J Haematol.

[20] Moorman AV, Roman E, Cartwright RA, Morgan GJ (2002). Patients entered into MRC AML trials are biologically representative of the totality of the disease in the UK. Clin Lab Haematol.

[21] Lehmann S, Ravn A, Carlsson L, Antunovic P, Deneberg S, Möllgård L, Rangert Derolf A, Stockelberg D, Tidefelt U, Wahlin A, Wennström L, Höglund M, Juliusson G (2011). Continuing high early death rate in acute promyelocytic leukemia: a population-based report from the Swedish Adult Acute Leukemia Registry. Leukemia.

[22] Rego EMR (2005). Development of the IC APL Project in Brazil. Haematologica Reports.

[23] Sanz MA, Lo CF, Martin G, Avvisati G, Rayon C, Barbui T, az-Mediavilla J, Fioritoni G, Gonzalez JD, Liso V, Esteve J, Ferrara F, Bolufer P, Bernasconi C, Gonzalez M, Rodeghiero F, Colomer D, Petti MC, Ribera JM, Mandelli F (2000). Definition of relapse risk and role of nonanthracycline drugs for consolidation in patients with acute promyelocytic leukemia: a joint study of the PETHEMA and GIMEMA cooperative groups. Blood.

[24] Gallagher RE, Willman CL, Slack JL, Andersen JW, Li YP, Viswanatha D, Bloomfield CD, Appelbaum FR, Schiffer CA, Tallman MS, Wiernik PH (1997). Association of PML-RAR alpha fusion mRNA type with pretreatment hematologic characteristics but not treatment outcome in acute promyelocytic leukemia: an intergroup molecular study. Blood.

[25] Douer D, Santillana S, Ramezani L, Samanez C, Slovak ML, Lee MS, Watkins K, Williams T, Vallejos C (2003). Acute promyelocytic leukaemia in patients originating in Latin America is associated with an increased frequency of the bcr1 subtype of the PML/RARalpha fusion gene. Br J Haematol.

[26] Dong S, Geng JP, Tong JH, Wu Y, Cai JR, Sun GL, Chen SR, Wang ZY, Larsen CJ, Berger R (1993). Breakpoint clusters of the PML gene in acute promyelocytic leukemia: primary structure of the reciprocal products of the PML-RARA gene in a patient with t(15;17). Genes Chromosomes Cancer.

[27] Uriarte MA, Zubillaga MNZ, Chacon AC, Bononi RB, Lombardi VL, Giordano HG, Fernandez IF, Manrique GM, Di Matteo CM, Pavlovsky SP, Martinez LM (2005). Genetic Characterization and Follow-up of MRD in Acute Promyelocytic Leukemia Patients from Argentina and Uruguay. Haematologica Reports.

[28] da Costa Moraes, Trompieri CA, Cavalcante NM, Felix FH (2008). Pediatric acute promyelocytic leukemia: all-transretinoic acid therapy in a Brazilian pediatric hospital. J Pediatr Hematol Oncol.

[29] Pagnano KB, de Carvalho Duarte G, Lorand-Metze I, Delamain MT, Miranda EC, De Souza CA (2010). Treatment outcome of acute promyelocytic leukemia with modified aida protocol. Adv Hematol.

[30] Leander C, Fu LC, Pena A, Howard SC, Rodriguez-Galindo C, Wilimas JA, Ribeiro RC, Haik B (2007). Impact of an education program on late diagnosis of retinoblastoma in Honduras. Pediatr Blood Cancer.

[31] Howard SC, Pedrosa M, Lins M, Pedrosa A, Pui CH, Ribeiro RC, Pedrosa F (2004). Establishment of a pediatric oncology program and outcomes of childhood acute lymphoblastic leukemia in a resource-poor area. JAMA.

[32] Howard SC, Marinoni M, Castillo L, Bonilla M, Tognoni G, Luna-Fineman S, Antillon F, Valsecchi MG, Pui CH, Ribeiro RC, Sala A, Barr RD, Masera G, MISPHO Consortium Writing Committee (2007). Improving outcomes for children with cancer in low-income countries in Latin America: a report on the recent meetings of the Monza International School of Pediatric Hematology/Oncology (MISPHO)-Part I. Pediatr Blood Cancer.

[33] Falini B, Flenghi L, Fagioli M, Lo Coco F, Cordone I, Diverio D, Pasqualucci L, Biondi A, Riganelli D, Orleth A, Liso A, Martelli MF, Pelicci PG, Pileri S (1997). Immunocytochemical diagnosis of acute promyelocytic leukemia (M3) with the monoclonal antibody PG-M3 (anti-PML). Blood.

[34] Sanz MA, Grimwade D, Tallman MS, Lowenberg B, Fenaux P, Estey EH, Naoe T, Lengfelder E, Büchner T, Döhner H, Burnett AK, Lo-Coco F (2009). Management of acute promyelocytic leukemia: recommendations from an expert panel on behalf of the European LeukemiaNet. Blood.

[35] Sanz MA, Montesinos P, Rayón C, Holowiecka A, de la Serna J, Milone G, de Lisa E, Brunet S, Rubio V, Ribera JM, Rivas C, Krsnik I, Bergua J, González J, Díaz-Mediavilla J, Rojas R, Manso F, Ossenkoppele G, González JD, Lowenberg B, PETHEMA and HOVON Groups (2010). Risk-adapted treatment of acute promyelocytic leukemia based on all-trans retinoic acid and anthracycline with addition of cytarabine in consolidation therapy for high-risk patients: further improvements in treatment outcome. Blood.

[36] Rego EM, Kim HT, Ruiz-Arguelles GJ, Uriarte R, Jacomo RH, Gutiérrez-Aguirre H, Melo RAM, Bittencourt R, Pasquini R, Pagnano KBB, Fagundes EM, Chauffaille MLLF, Chiattone C, Martinez L, Gomez-Almaguer D, Kwaan HC, Garcez-Eisele J, Gallagher RE, Niemeyer CM, Lowenberg B, Ribeiro RC, Lo-Coco F, Sanz MA (2009). Improving the Treatment Outcome of Acute Promyelocytic Leukemia in Developing Countries through International Cooperative Network. Report On the International Consortium On Acute Promyelocytic Leukemia Study Group. Blood (ASH Annual Meeting Abstracts).

